# A Novel Single-Test Approach for GDM Diagnosis: Identification and Prediction of High-Risk Postprandial Hyperglycemia

**DOI:** 10.3390/metabo16010027

**Published:** 2025-12-25

**Authors:** Hao Wu, Danqing Chen, Xue Li, Menglin Zhou, Qi Wu

**Affiliations:** 1 Women’s Hospital School of Medicine, Zhejiang University, Hangzhou 310027, China; 12118536@zju.edu.cn (H.W.); marlin_zhou@zju.edu.cn (M.Z.); 428102@zju.edu.cn (Q.W.); 2 Key Laboratory of Systems Health Science of Zhejiang Province, School of Life Science, Hangzhou Institute for Advanced Study, University of Chinese Academy of Sciences, Hangzhou 101408, China

**Keywords:** gestational diabetes mellitus, postprandial hyperglycemia, metabolic biomarkers, predictive model, ROC curve, nomogram

## Abstract

**Background:** Early prediction of gestational diabetes mellitus (GDM) remains a major clinical challenge, and the current oral glucose tolerance test (OGTT) is time-consuming and inconvenient for clinical routine. This study aimed to develop a novel predictive model for postprandial hyperglycemia GDM (pp-GDM) and postprandial glucose elevation using fasting serological and metabolic profiles. **Method:** We used High-Performance Liquid Chromatography-Mass Spectrometry (HPLC-MS) to analyze fasting plasma amino acid profiles at 24–28 weeks of gestation for 60 pp-GDM patients and 120 controls. Binary logistic regression model was constructed to identify potential biomarkers for pp-GDM prediction. **Results:** By incorporating amino acid indicators such as isoleucine, phenylalanine, threonine, and aspartate into the predictive model alongside traditional predictors (including BMI at sampling, fasting insulin, glycated hemoglobin, and uric acid), the overall predictive performance was significantly improved from 78.2% to 91.1%. A clinically practical nomogram for risk assessment was subsequently developed. **Conclusions:** This fasting metabolite-based model provides a reliable tool for early prediction of pp-GDM and postprandial hyperglycemia, which may reduce the need for OGTT and facilitate timely clinical decision making.

## 1. Introduction

Gestational diabetes mellitus (GDM) is a common clinical condition characterized by new-onset glucose intolerance during pregnancy. As the most prevalent metabolic disorder in pregnancy, GDM significantly increases the risk of adverse maternal and neonatal outcomes [1,2]. Current diagnostic criteria based on the one-step approach define GDM as fasting plasma glucose ≥ 5.1 mmol/L, 1-h postprandial glucose ≥ 10.0 mmol/L, or 2-h postprandial glucose ≥ 8.5 mmol/L [3,4]. While early detection of fasting hyperglycemia in pregnancy allows for timely intervention and improved outcomes, postprandial hyperglycemia, often asymptomatic and requiring Oral glucose tolerance test (OGTT) for diagnosis [5,6], is frequently underrecognized. Notably, postprandial hyperglycemia GDM (pp-GDM) accounts for over 90% of dysglycemia cases in pregnancy and is associated with more profound impacts on pancreatic β-cell function and vascular health [1,7,8]. Poor patient compliance with the OGTT—due to discomfort from repeated venipuncture and the logistical burden of prolonged clinic visits—often leads to missed diagnosis of postprandial hyperglycemia. Thus, this study aims to develop a method for identifying individuals at high risk of postprandial dysglycemia using only fasting metabolic measurements [9].

Emerging evidence suggests that metabolic disturbances in GDM extend beyond glucose homeostasis [10]. Dysregulation of specific amino acids and lipids—closely linked to mitochondrial tricarboxylic acid cycle (TCA cycle) flux—has been increasingly implicated in GDM pathophysiology [11]. With advances in clinical metabolomics, we can now identify clinically actionable biomarkers reflecting underlying metabolic dysfunction [12]. Many of these biomarkers originate from amino acids and have been shown to disrupt both energy metabolism and insulin signaling, contributing to hyperglycemia and insulin resistance. Most existing GDM prediction models rely on conventional risk factors and have limited clinical utility due to modest performance [13,14]. Furthermore, current models rarely account for the distinct mechanisms underlying fasting and postprandial hyperglycemia.

To address these gaps, we focused specifically on postprandial hyperglycemia—the most common phenotype of GDM—and conducted a comprehensive metabolic evaluation in mid-pregnancy. Using fasting samples from pp-GDM patients and glucose-tolerant controls, we measured glucose, insulin, glycated hemoglobin (HbA1c), and performed broad metabolic profiling including amino acids and lipids quantification. We targeted common, stable amino acids present in human metabolism, with a focus on species previously implicated in oxidative stress and insulin resistance, such as glutamine, branched-chain amino acids, and aromatic amino acids. From these, we selected readily measurable fasting metabolites to develop a practical prediction model for identifying women at high risk of postprandial dysglycemia, with the goal of enabling earlier intervention and improved clinical management.

## 2. Materials and Methods

### 2.1. Study Design and Participants

This population-based, retrospective case–control study analyzed fasting serum samples from pregnant women aged 25–45 years. We consecutively recruited women in their mid-trimester (24–28 weeks of gestation) who attended routine prenatal check-ups at the Women’s Hospital, Zhejiang University School of Medicine, between 1 January 2023 and 1 January 2024. All participants fasted for 8–12 h prior to sample collection.

Diagnosis of GDM was based on the one-step method, with a positive case defined as any subject meeting or exceeding the threshold for fasting plasma glucose (FPG > 5.1 mM), 1-h glucose (>10.0 mM), or 2-h glucose (>8.5 mM).

This study was designed to investigate GDM patients with isolated postprandial hyperglycemia. Consequently, individuals exhibiting abnormal fasting hyperglycemia were not included in the study population. The exclusion criteria were as follows: (1) abnormal fasting blood glucose (>5.1 mM or <3.5 mM); (2) use of medications affecting glucose metabolism (e.g., oral hypoglycemic agents, insulin, or corticosteroids); (3) thyroid dysfunction; (4) comorbid rheumatic or autoimmune diseases; (5) hepatic or renal dysfunction; (6) twin or multiple pregnancies; and (7) dietary modifications for specific purposes.

During the study period, 124 fasting blood samples from women with GDM were initially collected. After screening, 34 subjects who did not complete the OGTT, 19 subjects with missing key clinical data or incomplete serological tests, and 11 subjects who declined to participate were excluded. Consequently, 60 pp-GDM cases were included for subsequent amino acid and lipid profiling. These cases were matched at a 1:2 ratio with 120 healthy pregnant women in their mid-trimester, based on age, gravidity, and parity. According to PASS 2021 calculations, the statistical power of this experimental design is greater than 0.80.

This study was designed according to the Strengthening the Reporting of Observational Studies in Epidemiology (STROBE statement) and was approved by the Ethics Committee of the Women’s Hospital, Zhejiang University School of Medicine (IRB-20230160-R). Written informed consent was obtained from all participants.

### 2.2. Data Collection and Biochemical Measurements

Anthropometric data (including pre-pregnancy weight, gestational weight gain, and height), gestational age at sample collection, lifestyle history, and medication history were retrieved from the hospital’s electronic medical record system.

All serum biochemical parameters were measured in the hospital’s clinical laboratory. Fasting blood glucose (FBG), 1-h OGTT, 2-h OGTT, fasting insulin (FINS), triglycerides (TG), total cholesterol (TC), high-density lipoprotein (HDL), low-density lipoprotein (LDL), HbA1c, and other biochemical indices were determined by chemiluminescent microparticle immunoassay (UniCel DXI 800; Beckman Coulter, Brea, CA, USA). The homeostasis model assessment of insulin resistance (HOMA-IR) index was calculated as follows: HOMA-IR = [Fasting Glucose (mmol/L) × Fasting Insulin (μIU/mL)]/22.5.

### 2.3. Quantification of Amino Acid Profiles

Quantitative analysis of amino acids was performed using a Scientific TSQ9000 triple quadrupole LC-MS/MS system (Thermo Fisher, Shanghai, China). The chromatographic conditions were as follows: a DB-5MS capillary column (30 m × 0.25 mm × 0.25 μm; Agilent J&W Scientific, Beijing, China); high-purity helium (≥99.999%) as the carrier gas at a flow rate of 1.2 mL/min; injector temperature at 260 °C; injection volume of 1 μL in splitless mode with a 4-min solvent delay. The oven temperature program was set as: initial hold at 50 °C for 0.5 min, ramp to 125 °C at 15 °C/min and hold for 2 min, then to 210 °C at 8 °C/min and hold for 2 min, subsequently to 270 °C at 11 °C/min and hold for 1 min, and finally to 305 °C at 25 °C/min with a 3-min hold. Mass spectrometry conditions utilized an electron impact (EI) ion source at 300 °C, a transfer line temperature of 280 °C, and selective reaction monitoring (SRM) mode with a mass scan range of *m*/*z* 40–600. A total of 19 amino acids were included in our research. Detailed information is provided in Supplementary Material S1.

### 2.4. Statistical Analysis

Continuous variables are presented as mean ± standard deviation. Inter-group comparisons were performed using independent samples *t*-test or the Kruskal–Wallis non-parametric test, based on normality testing results. Categorical variables are presented as counts (percentages) and were compared using the chi-square test. Amino acid levels were normalized to the standard deviation and expressed as Z-scores. Bonferroni correction for multiple comparisons was applied.

To identify independent predictors of pp-GDM characterized by isolated postprandial hyperglycemia, potential influencing variables were included as independent variables in step forward binary logistic regression models to calculate odds ratios (ORs) with 95% confidence intervals (95% CIs). Predictive models for pp-GDM characterized by elevated postprandial glucose were constructed. To rank the predictors, we utilized the random forest method to quantify the relative importance of each factor. Stratified logistic regression models were built by combining metabolites from different sources, and the predictive performance of these models was evaluated using Receiver Operating Characteristic (ROC) curves and the Area Under the Curve (AUC).

To explore the contribution of individual predictors to postprandial glucose levels and glucose area under the curve (AUC-G), Pearson correlation and adjusted partial correlation analyses (controlling for age, gravidity, parity, smoking, and alcohol history) were employed to identify relevant variables. Also, multiple linear regression analysis was used to identify independent risk factors for abnormal postprandial glucose changes.

A two-sided *p*-value < 0.05 was considered statistically significant for all analyses. All statistical analyses were conducted using IBM SPSS Statistics for Windows, Version 26.0 (IBM Corp., Armonk, NY, USA). Sample size was determined a priori using PASS, Version 2021 (NCSS, LLC., Kaysville, UT, USA), Figures were generated using GraphPad Prism, Version 9.5.0 (GraphPad Software, Boston, MA, USA).

## 3. Results

A total of 180 participants, comprising 60 pp-GDM cases and 120 matched healthy controls (1:2 ratio based on age, gravidity, and parity), were ultimately included in the analysis. The baseline characteristics and laboratory parameters of the two groups are detailed in Table 1.

### 3.1. Participant Characteristics and Metabolic Profiles

As shown in Table 1, pre-pregnancy body mass index (BMI) was significantly higher in the pp-GDM group, and this difference persisted at the time of sampling. Consequently, the proportion of individuals who were overweight or obese was significantly greater in the pp-GDM group. No significant differences were observed between the two groups regarding history of adverse pregnancy outcomes, gestational week at sampling, or weight gain during mid-pregnancy.

Regarding glucose metabolism, the pp-GDM group exhibited significantly elevated levels of fasting blood glucose (FBG), 1-h and 2-h oral glucose tolerance test (OGTT) values, glycated hemoglobin (HbA1c), and homeostasis model assessment of insulin resistance (HOMA-IR). Among the lipid profiles, only triglyceride (TG) levels were significantly higher in the pp-GDM group. Furthermore, although uric acid levels in both groups fell within the normal reference range, the pp-GDM group had significantly higher uric acid levels than the control group.

### 3.2. Altered Fasting Amino Acid Profiles in pp-GDM

**Figure 1:**
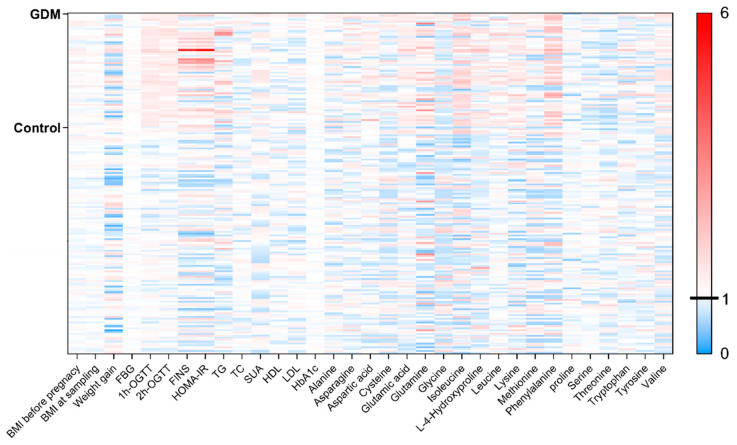
Normalized visualization based on the mean values of control group parameters.

The fasting serum amino acid levels, quantified by liquid chromatography-mass spectrometry (LC-MS), are presented in Table 2 and Figure 1. Compared to the control group, the pp-GDM group showed varying degrees of elevation in multiple amino acids, including alanine, aspartic acid, asparagine, glutamic acid, glutamine, hydroxyproline, lysine, phenylalanine, valine, and all branched-chain amino acids. Notably, the increases in glutamine, isoleucine, leucine, phenylalanine, and valine were particularly pronounced.

Standardization was performed by calculating deviations from control group mean values for each parameter.

### 3.3. Development and Evaluation of a pp-GDM Risk Prediction Model

**Figure 2:**
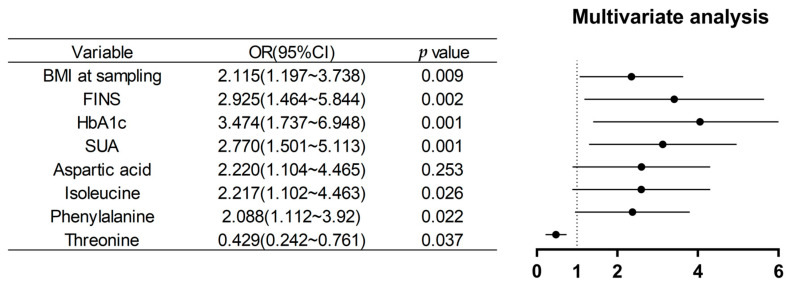
Risk factor exposure analysis for GDM.

**Figure 3:**
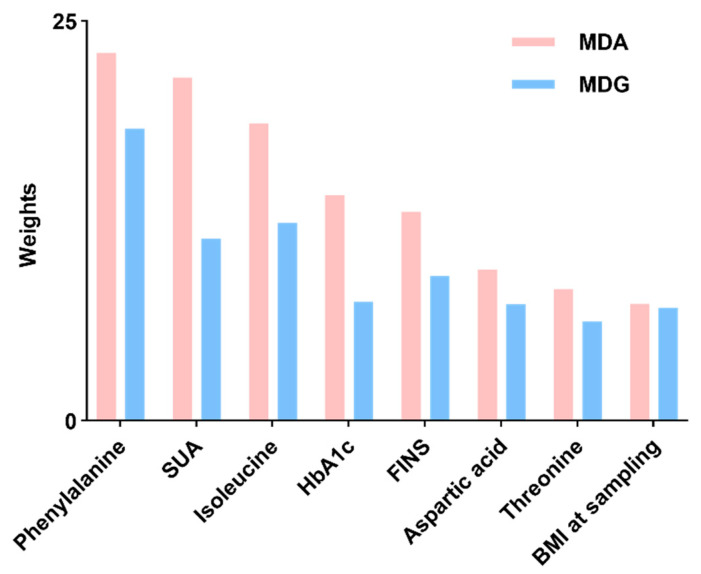
Assessment of Predictor Variable Importance.

To develop a predictive model, a binary logistic regression analysis was conducted with pp-GDM as the dependent variable, incorporating all significant influencing factors (*p* < 0.05). Binary logistic regression identified BMI at sampling, f (FINS, HbA1c, SUA, isoleucine, and phenylalanine as independent risk factors for GDM, whereas threonine served as a protective factor (Figure 2 and Figure 3). Random forest analysis ranked phenylalanine, valine, and uric acid as the most important contributors.

Independent variables: Parameters related to carbohydrate metabolism, lipid metabolism, and amino acid metabolism. Analytical method: Stepwise forward binary logistic regression to assess associations between exposures and pp-GDM occurrence. OR (95% CI): Odds ratio with 95% confidence interval, quantifying the strength and precision of exposure-outcome associations. OR < 1 suggests protective factor; OR > 1 indicates risk factor. Horizontal lines represent 95% confidence intervals.

Goodness-of-fit test: The Hosmer–Lemeshow test indicated good model fit (χ^2^ = 5.82, *p* = 0.176 > 0.05). Predictive performance: Control group accuracy: 95% (95% CI: 86.1–98.3%); pp-GDM group accuracy: 83.3% (95% CI: 72.4–90.5%); Overall accuracy: 91.1% (95% CI: 85.7–94.6%).

Assessment of feature importance via random decision forests, employing Mean Decrease Accuracy (MDA) and Mean Decrease Gini (MDG) to evaluate the impact of factor fluctuations on model performance.

**Figure 4:**
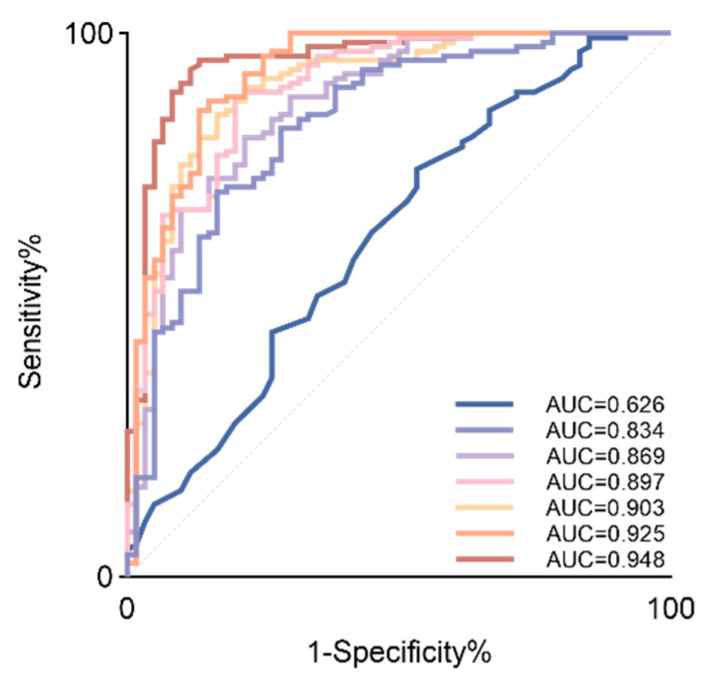
ROC curves for predictive models employing different combinations of risk factors.

**Figure 5:**
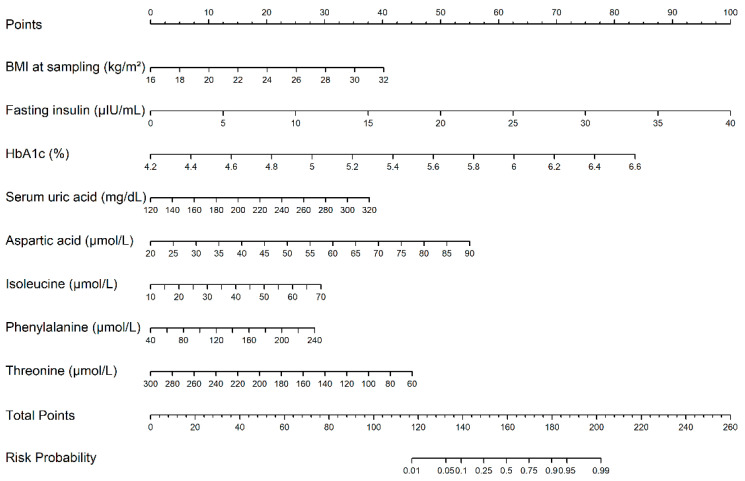
A Prediction Nomogram for Mid-Trimester Postprandial Hyperglycemia Using Independent Risk Factors.

We further evaluated the predictive performance for pp-GDM using parameters from different categories (including glucose, lipids, uric acid, amino acids, and body weight metrics) (Table 3, Figure 4). The model using BMI alone yielded the weakest predictive accuracy. Notably, integrating it with biochemical parameters failed to surpass the predictive efficacy of the stand-alone amino acid profile. The comprehensive model integrating amino acids, body weight, and biochemical parameters demonstrated the best performance, achieving an AUC of 0.948 (95% CI: 0.911–0.985). Based on sampling-time BMI, fasting insulin (FINS), HbA1c, uric acid, isoleucine, phenylalanine and threonine, we constructed a prognostic nomogram for pp-GDM risk assessment, which provides a visual tool for estimating the probability of abnormal postprandial glucose elevation (Figure 5).

We conducted a single-blind evaluation of the predictive model using an external sample cohort. The assessors, blinded to the OGTT results, applied the model to predict postprandial GDM (pp-GDM). The model demonstrated a sensitivity of 91% and a specificity of 100%. Based on these findings, individuals with a predicted risk ≥ 95% were classified as high-risk for pp-GDM. To align with the actual prevalence of GDM in our population, we subsequently adjusted the risk threshold to ≥90% for identifying high-risk individuals who would be enrolled in specialized gestational management. Detailed information is provided in Supplementary Material S2.

Based on different data types, we constructed multiple predictive models: Model 1: Body weight indicators; Model 2: Biochemical indicators; Model 3: Biochemical indicators & body weight indicators; Model 4: Amino acid indicators & body weight indicators; Model 5: Amino acid indicators only; Model 6: Amino acid indicators & biochemical indicators; Model 7: All indicators. ROC curves were generated based on these models, and model performance was evaluated using the area under the curve (AUC).

### 3.4. Determinants of Postprandial Glucose Levels and Glucose Aera Under the Curve

The seven independent risk factors were incorporated into multiple linear regression models to assess their predictive power for 1h-OGTT, 2h-OGTT, and the area under the glucose curve (AUC-G) (Table 4). While the model predicting 1h-OGTT based on fasting indices showed a reasonable fit (adjusted R^2^ = 0.596), the predictive efficacy for 2h-OGTT was relatively modest (adjusted R^2^ = 0.466). Nevertheless, the model still achieved a satisfactory prediction for the overall glycemic load (AUC-G, adjusted R^2^ = 0.589).

## 4. Discussion

This study represents the first attempt to integrate routine prenatal check-up parameters with a panel of primary energy metabolites, quantified from a single serum sample, to predict the probability of GDM characterized by postprandial hyperglycemia. We identified BMI at sampling, FINS, HbA1c, SUA, and several amino acids as independent risk factors for this GDM subtype. Based on these factors, we developed a high-performance prediction model (AUC = 0.948) and a clinically applicable risk assessment nomogram.

### 4.1. Clinical Implication of Prediction of Postprandial Hyperglycemia in GDM

With information obtained from a single fasting serum sample, our model provides a precise and clinically applicable tool for identifying pp-GDM patients at risk of postprandial hyperglycemia.

The pathophysiology of GDM with postprandial hyperglycemia differs from that of the fasting hyperglycemia subtype [15]. The latter is primarily associated with decreased hepatic insulin sensitivity, whereas the former involves more severe systemic insulin resistance and inadequate compensatory insulin secretion by pancreatic β-cells [16]. In our cohort, over 85% of GDM cases were characterized by isolated postprandial hyperglycemia. This subtype is closely linked to an increased risk of adverse pregnancy outcomes and long-term metabolic diseases [3], underscoring the importance of its specific investigation for understanding the pathogenesis of most GDM cases [17].

The predictive performance of our model (AUC = 0.948) compares favorably with previous studies. Compared with our model, previously reported GDM prediction models exhibit certain limitations in either performance or generalizability. For instance, a Southeast Asian model (AUC = 0.81), while incorporating multiple biomarkers [18], may have limited applicability to other ethnic groups. A model derived from a large Chinese cohort (AUC = 0.77) relied solely on routine clinical variables, resulting in suboptimal predictive power [19]. Another smaller study (NGDM = 20) reported a higher AUC (0.87) [20], but the limited sample size raises concerns about potential overfitting, and its model included non-standardized measurements such as tissue plasminogen activator. Limitations of these models include suboptimal predictive power or reliance on biomarkers not routinely assessed, thereby restricting their clinical utility. In contrast, our model focuses on stable small molecules central to energy metabolism (carbohydrates, lipids, amino acids) and utilizes data obtainable from a single, routine clinical blood draw. This approach ensures high predictive accuracy while maintaining strong potential for clinical translation.

### 4.2. Obesity, Insulin Resistance, and Uric Acid as Major Drivers of pp-GDM Risk

Obesity is a well-established initiator of GDM. Our study confirmed that the pp-GDM group had consistently higher BMI from pre-pregnancy through mid-pregnancy, with a significantly greater proportion of overweight/obese individuals. A state of chronic high BMI is often accompanied by low-grade adipose tissue inflammation [21], where released pro-inflammatory cytokines such as Tumor necrosis factor-α (TNF-α) and Interleukin-6 (IL-6) can directly interfere with insulin signaling pathways, exacerbating insulin resistance [22,23].

Within our model, elevated fasting insulin is an early marker of insulin resistance and β-cell compensation. HbA1c is closely correlated with postprandial glucose excursions, and its elevation suggests that postprandial glucose may account for over 70% of the daily glycemic load [15,24,25]. Of particular note, although serum uric acid levels are generally low in pregnancy, we identified it as a strong predictor for pp-GDM (OR = 2.77 per SD) [26]. This aligns with recent evidence indicating that, even within the adjusted normal range, higher uric acid levels are associated with increased pp-GDM risk and greater postprandial glucose fluctuations, highlighting it as a previously overlooked significant indicator.

### 4.3. The Unique Predictive Value and Potential Mechanisms of Amino Acids

Targeted metabolomics revealed that aspartate, isoleucine, and phenylalanine served as independent risk factors for GDM, whereas threonine exhibited a protective effect. This finding is partially supported by existing literature. A nested prospective cohort study in Singapore monitored amino acid dynamics across early (12–14 weeks) and mid-pregnancy (24–28 weeks), reporting sustained positive correlations between glutamate, aspartate, and glutamine and subsequent GDM development [2]. Another study, analyzing fasting mid-pregnancy amino acid pathways, indicated an inverse association between the glycine-serine-threonine metabolic pathway and GDM risk [14,27].

Previous research suggests that postprandial hyperglycemia arises from progressive β-cell dysfunction and peripheral insulin resistance, often attributed to oxidative stress-induced decompensation of pancreatic islet function and disruption of insulin signaling pathways during pregnancy [28]. The identified amino acids may influence these pathways through distinct mechanisms. Aspartate acid has been shown to suppress AMP-activated protein kinase (AMPK) activity, promoting lipid accumulation in pancreatic β-cells [24,29]. Phenylalanine can inhibit tyrosine kinase activity of the insulin receptor β-subunit via aminoacylation, thereby impairing downstream insulin signal transduction in peripheral tissues [30]. Dietary intervention studies in animals demonstrate that reduced isoleucine intake significantly improves hepatic insulin sensitivity and enhances postprandial glucose storage as glycogen [31]. In contrast, the protective role of threonine may be attributed to its function as a superoxide dismutase activator and its capacity to scavenge reactive oxygen species, thereby mitigating oxidative stress [23].

Although fasting amino acid levels generally decrease during pregnancy due to fetal demands and hemodilution, and dietary intake introduces variability, homeostatic regulation—primarily through the TCA cycle—maintains relative stability in most circulating amino acids [11,16]. However, conditions such as obesity, smoking, and pre-existing glucose intolerance exacerbate oxidative stress and insulin resistance. These factors can dysregulate TCA cycle intermediates and contribute to the abnormal accumulation of specific amino acids, which are increasingly recognized as key contributors to the pathogenesis of GDM [10].

### 4.4. Study Strengths

The primary strength of this study lies in the successful development of a high-performance predictive model for postprandial hyperglycemia-defined GDM, based on primary metabolites and routine clinical parameters from a single fasting serum sample. This approach utilizes biological samples routinely collected during prenatal check-ups, thereby significantly expanding the clinical utility of fasting blood tests without substantially increasing healthcare costs or patient burden. It provides an efficient and convenient tool for middle-stage risk stratification of pp-GDM.

### 4.5. Study Limitations and Future Perspectives

Our study has several limitations that should be considered. First, the predictive model was developed and validated specifically for postprandial glucose levels in mid-pregnancy; its applicability for early pregnancy screening has not been evaluated. Second, the model was derived from a single-center study with a relatively small sample size. Therefore, its generalizability requires further validation in larger, multi-center prospective cohorts. The proposed multi-amino acid assay adds only an estimated $7 per patient to the cost of the standard OGTT. Furthermore, it streamlines the clinical workflow by requiring fewer blood samples and shortening the protocol by about two hours. Its translation into routine practice, however, is contingent upon the local availability of quantitative amino acid analysis capabilities.

## 5. Conclusions

This study demonstrates that a predictive model integrating fasting plasma amino acid profiles with routine clinical parameters significantly enhances the early identification of women at high risk for pp-GDM. The incorporation of specific amino acids—isoleucine, phenylalanine, threonine, and aspartate—into a model alongside traditional markers (BMI, FINS, HbA1c, and SUA) improved the predictive accuracy to 91.1%, representing a substantial advance over models relying solely on conventional factors. The developed nomogram translates these complex biomarkers into a practical, visual tool for clinicians, enabling rapid risk stratification during the pivotal 24–28 week gestation window using only a single fasting blood sample.

## Figures and Tables

**Table 1:** Baseline Characteristics and Routine biochemical tests of Study Participants Stratified by pp-GDM Status.

**Variable**	**pp-GDM (n = 60)**	**Control (n = 120)**	***p*** **Value**
Age, years	32.88 ± 4.89	32.23 ± 3.82	0.369
Smoking	0	2	0.989
Alcohol abuse	2	5	0.997
Gestational age at sampling, weeks	25.6 ± 1.48	25.2 ± 1.47	0.089
Pre-pregnancy BMI, kg/m^2^	21.64 ± 3.17	20.19 ± 2.17	0.002 **
Overweight	8	4	
Obesity	1	0	
BMI at sampling, kg/m^2^	24.84 ± 2.94	23.52 ± 2.55	0.004 **
Gestational weight gain, kg	10.88 ± 4.85	10.36 ± 4.21	0.481
Gravidity			0.757
1	20	35	
2	15	36	
≥3	25	109	
Parity			0.576
0	33	69	
1	24	48	
≥2	3	63	
Adverse pregnancy			0.281
0	30	70	
1	21	35	
≥2	9	15	
FBG, mM	4.32 ± 0.32	4.14 ± 0.27	0.001 ***
1h-OGTT, mM	10.27 ± 1.3	7.46 ± 1.23	0.001 ***
2h-OGTT, mM	8.8 ± 1.16	6.55 ± 0.94	0.001 ***
FINS, μIU/mL	10.54 ± 5.36	7.44 ± 2.46	0.001 ***
* HOMA-IR	2.05 ± 1.12	1.37 ± 0.48	0.001 ***
HbA1c, %	5.13 ± 0.36	4.82 ± 0.27	0.001 ***
TG, mM	2.77 ± 1.23	2.1 ± 0.66	0.001 ***
TC, mM	6.41 ± 1.31	6.49 ± 1.02	0.679
HDL, mM	2.05 ± 0.33	2.07 ± 0.36	0.739
LDL, mM	2.91 ± 0.74	2.94 ± 0.65	0.799
SUA, μM	227.67 ± 52.49	196.17 ± 45.13	0.001 ***

Continuous variables were assessed for normality using Shapiro–Wilk tests. Normally distributed variables were compared using Student’s *t*-tests, while non-normally distributed variables were analyzed with Mann–Whitney U tests. Continuous variables were presented as mean ± standard deviation (Mean ± SD); pp-GDM: Gestational Diabetes Mellitus with postprandial hyperglycemia; FBG: Fasting blood glucose; OGTT: Oral glucose tolerance test; FINS: Fasting insulin; HOMA-IR: Homeostatic Model Assessment of Insulin Resistance; TG: Total triglyceride; TC: Total cholesterol; HDL: High-density lipoprotein; LDL: Low-density lipoprotein; HbA1c: Glycosylated Hemoglobin, Type A1C; SUA: Serum Uric Acid. *: *p* < 0.05, ** *p* < 0.01; *** *p* < 0.001.

**Table 2:** Quantitative detection of fasting amino acids based on LC-MS.

Variable	pp-GDM (n = 60)	CONTROL (n = 120)	*p* Value
Alanine	323.66 ± 86.43	297.57 ± 73.77	0.04 *
Asparagine	64.99 ± 18.41	56.86 ± 13.69	0.001 ***
Aspartic acid	42.17 ± 10.75	35.56 ± 7.73	0.001 ***
Cysteine	158.22 ± 48	153.87 ± 51.5	0.59
Glutamic acid	119.23 ± 26.15	92.42 ± 23.09	0.001 ***
Glutamine	1699.29 ± 699.18	1216 ± 688.41	0.001 ***
Glycine	155.43 ± 56.16	156.58 ± 63.63	0.91
Isoleucine	45.24 ± 13.22	29.53 ± 12.66	0.001 ***
L-4-Hydroxyproline	15.02 ± 4.29	12.07 ± 4.14	0.001 ***
Leucine	158.75 ± 41.14	128.75 ± 24.19	0.001 ***
Lysine	667.55 ± 145.4	506.67 ± 190.41	0.001 ***
Methionine	17.91 ± 5.26	17.05 ± 6.23	0.36
Phenylalanine	141.32 ± 46.1	85.31 ± 36.39	0.001 ***
proline	123.61 ± 24.41	124.43 ± 22.41	0.82
Serine	121.81 ± 31.02	128.32 ± 20.07	0.09
Threonine	142.31 ± 43.89	159.29 ± 39.15	0.01 **
Tryptophan	687.99 ± 227.75	632.22 ± 161.26	0.06
Tyrosine	62.05 ± 18.47	61.8 ± 14.84	0.92
Valine	182.8 ± 43.74	147.18 ± 43.32	0.001 ***

Continuous variables were presented as mean ± standard deviation (Mean ± SD). *: *p* < 0.05, ** *p* < 0.01; *** *p* < 0.001. Unit: Μm.

**Table 3:** ROC curves for predictive models employing different combinations of risk factors; ** *p* < 0.01; *** *p* < 0.001.

	AUC (95%CI)	*p* Value
Model 1	0.626 (0.538~0.715)	0.006 **
Model 2	0.834 (0.770~0.899)	0.001 ***
Model 3	0.869 (0.811~0.927)	0.001 ***
Model 4	0.897 (0.848~0.948)	0.001 ***
Model 5	0.903 (0.853~0.952)	0.001 ***
Model 6	0.925 (0.879~0.971)	0.001 ***
Model 7	0.948 (0.911~0.985)	0.001 ***

**Table 4:** Multivariable Linear Regression Modeling of Postprandial Glucose Using Fasting Parameters.

	β (95%CI)	*p* Value
1h-OGTT (R^2^ = 0.596)		
FINS	0.133 (0.061~0.425)	0.009 **
HbA1c	0.213 (0.199~0.566)	0.001 ***
SUA	0.128 (0.054~0.411)	0.011 ***
Isoleucine	0.374 (0.465~0.917)	0.001 ***
Phenylalanine	0.271 (0.271~0.724)	0.001 ***
Threonine	−0.178 (−0.501~−0.150)	0.001 ***
2h-OGTT (R^2^ = 0.366)		
BMI at sampling	0.196 (0.105~0.469)	0.002 **
FINS	0.111 (−0.022~0.348)	0.083
HbA1c	0.271 (0.207~0.578)	0.001 ***
Isoleucine	0.142 (−0.018~0.440)	0.071
Phenylalanine	0.223 (0.100~0.558)	0.005 **
Threonine	−0.152 (−0.401~−0.045)	0.014 *
AUC-G (R^2^ = 0.589)		
BMI at sampling	0.088 (−0.030~0.458)	0.085
FINS	0.140 (0.091~0.591)	0.008 **
HbA1c	0.247 (0.345~0.842)	0.001 ***
SUA	0.121 (0.053~0.536)	0.017 *
Isoleucine	0.329 (0.505~1.118)	0.001 ***
Phenylalanine	0.264 (0.339~0.956)	0.001 ***
Threonine	−0.179 (−0.675~−0.196)	0.001 ***

AUC-G: Area Under the Curve for Glucose; The multiple regression model revealed significant associations with postprandial glucose concentrations (all timepoints *p* < 0.05). These relationships persisted after adjustment for potential confounders (parity, gravidity, age, and lifestyle factors) in partial correlation analysis. *: *p* < 0.05, ** *p* < 0.01; *** *p* < 0.001.

## Data Availability

The data provided in this study can be obtained from the corresponding author, as some of the data are still involved in unpublished research.

## Supplementary Materials

The following supporting information can be downloaded at: https://www.mdpi.com/article/10.3390/metabo16010027/s1, Supplementary Material S1: Standard curve for quantitative detection of amino acids; Supplementary Material S2: Test set for evaluating model performance.
